# E3 ligase Praja1 mediates ubiquitination and degradation of microtubule‐associated protein tau

**DOI:** 10.1111/febs.70303

**Published:** 2025-11-03

**Authors:** Shiho Aoki, Wataru Onodera, Akihiko Takashima, Kotaro Kawasaki, Kazuki Imadegawa, Hikaru Kurahashi, Mizuho Oishi, Toru Asahi, Yoshiyuki Soeda

**Affiliations:** ^1^ Faculty of Science and Engineering Waseda University Tokyo Japan; ^2^ Faculty of Science, Department of Life Science, Laboratory for Alzheimer's Disease Gakushuin University Tokyo Japan; ^3^ Research Organization for Nano & Life Innovation Waseda University Tokyo Japan; ^4^ Present address: Department of Physiology and Anatomy Nihon University School of Pharmacy Chiba Japan

**Keywords:** E3 ubiquitin ligase, Gene duplication, Molecular evolution, Neofunctionalization, Praja family, Praja1, Substrate specificity, Tau

## Abstract

The RING‐H2 type E3 ligase Praja family is composed of E3 ubiquitin–protein ligases Praja1 and Praja2, which promote the degradation of substrates through the ubiquitin–proteasome system. Both paralogs contribute to neuronal maturation and differentiation, indicating a significant role in the nervous system. Aggregation‐prone proteins associated with neurodegenerative diseases, including TAR DNA‐binding protein 43 (TDP‐43) and α‐synuclein, are degraded and/or suppressed by Praja1. Furthermore, the expression level of the microtubule‐associated protein tau (*MAPT*) gene, which is frequently mutated in Alzheimer's disease, is regulated by Praja2. Although the Praja family has been shown to recognize various aggregation‐prone proteins as substrates, it has not been determined whether tau, a key protein that aggregates in tauopathies, is also recognized by Praja proteins. In this study, we show that Praja1, but not Praja2, recognizes tau as a candidate substrate. We observed that the tau protein level in human neuroblastoma SH‐SY5Y cells decreased depending on the E3 ligase activity of Praja1. Furthermore, the *in vivo*/*in vitro* ubiquitination assay showed that Praja1 ubiquitinates tau, indicating that it is a target substrate. Next, by combining ancestral sequence reconstruction and mutational analysis, we revealed that the Praja1–tau interaction began just after the duplication of the Praja family in the common ancestor of placentals. Lastly, to test whether this interaction is disrupted under pathological conditions, P301L tau was introduced, resulting in a degradation similar to that of wild‐type tau. These results reveal an unidentified mechanism of tau proteostasis by Praja1 and may provide insight into the pathogenesis of neurodegenerative diseases, including tauopathy.

## Graphical abstract

The Praja family of RING‐H2 type E3 ligases is composed of E3 ubiquitin–protein ligases Praja1 and Praja2, which promote the degradation of substrates through the ubiquitin–proteasome system. We show that Praja1, but not its paralogue Praja2, recognizes and ubiquitinates tau protein for proteasomal degradation. Efficient proteasomal degradation of aggregation‐prone proteins, such as tau, is essential for neuroprotection. This newly identified function of Praja1 in tau degradation suggests its role in protein quality control, which may provide insights into the pathogenesis of tauopathies.

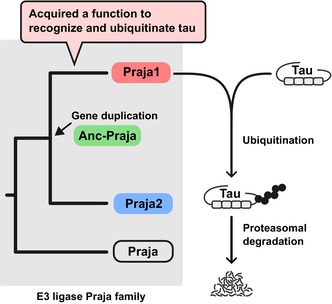

**Abbreviations**
AD: Alzheimer's disease
ALS: amyotrophic lateral sclerosis
BiFC: Bimolecular fluorescence complementation
FTDP: frontotemporal dementia
FTLD: frontotemporal lobar degeneration
GRASP: graphical representations of ancestral sequence predictions
MAPT: microtubule‐associated protein tau
PD: Parkinson's disease
TDP‐43: TAR DNA‐binding protein 43

## Introduction

The Praja family has been identified as a RING‐H2 type E3 ligase that functions in the ubiquitin–proteasome system to maintain intracellular proteostasis through substrate degradation [1, 2, 3]. In humans, this family consists of two paralogs, Praja1 and Praja2. Our previous study showed that these paralogs duplicated during evolution to placentals and that Praja1, in particular, may have functionally differentiated from Praja2 due to its increase in the evolutionary rate immediately after duplication [2].

The Praja family is expressed ubiquitously, with prevalence in brain regions such as the cerebellum and frontal cortex [1]. The physiological function of Praja is thought to be associated with neuronal morphology, and positive and negative regulation of neurite outgrowth has been reported [4, 5]. Studies indicate that Praja1 and Praja2 are associated with neuronal diseases. For example, TDP‐43, a major component of cytoplasmic aggregates present in amyotrophic lateral sclerosis (ALS) and frontotemporal lobar degeneration (FTLD), is a substrate of Praja1, and their interaction results in the inhibition of its phosphorylation and aggregation [6, 7]. Additionally, aggregation of proteins, including FUS, SOD1, α‐synuclein, ataxin‐3, and huntingtin polyglutamine proteins, is inhibited by Praja1 [8, 9]. Similarly, in human brains of AD patients, it was found that the expression of Praja2 gene expression was lower compared to the normal control [10].

Tau, encoded by the *MAPT* gene, is mainly involved in axonal transport and synaptic plasticity and is expressed in the central and peripheral nervous systems [11]. In neurons, it is primarily localized in the cytoplasm and regulates neuronal morphology by binding to and stabilizing microtubules [12, 13]. Alternative splicing of *MAPT* transcripts generates six major isoforms of human tau, and an imbalance between isoform expression or misfolding is observed in several neurodegenerative diseases, such as AD, Parkinson's disease (PD), and frontotemporal dementia (FTDP), all termed tauopathies [14, 15, 16].

Currently, it is unclear whether the Praja family recognizes tau as a substrate, along with other aggregation‐prone proteins. Clarifying this protein interaction may provide novel insights into the mechanisms of maintaining tau protein homeostasis disrupted in tauopathy. Here, we found reduced tau protein levels when Praja1, but not Praja2, was overexpressed in human neuroblastoma SH‐SY5Y cells. Knockdown of Praja1 resulted in an increase in endogenous tau level. By combining pharmacological, mutagenesis, and *in vivo*/*in vitro* ubiquitination assays, it was indicated that tau is recognized by Praja1 and is ubiquitinated and degraded as a candidate substrate. For its recognition, Praja1 requires a deletion mutation that occurred during its evolution in the common ancestor of placentals, as the artificial deletion of the region from Praja2 enabled it to recognize tau. Finally, we showed that the FTDP tau mutation P301L, which often disrupts the interaction between the physiological binding partners of tau, is recognized and degraded by Praja1. Our study provides a potential mechanism of tau proteostasis regulated by the E3 ligase Praja1, which may be particularly valuable in diseases such as tauopathy, in which tau proteostasis is disrupted.

## Results

### Praja1 regulates tau stability in human neuroblastoma SH‐SY5Y cells

**Fig. 1:**
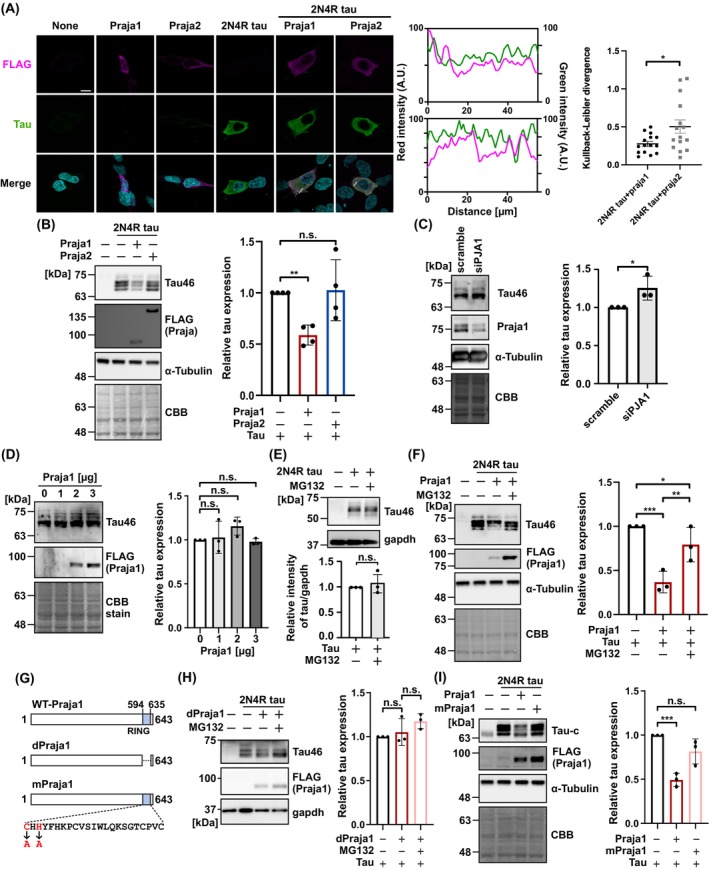
Praja1 negatively regulates tau in an E3 ligase activity‐dependent manner in SH‐SY5Y cells. All experiments were performed 16–24 h after plasmid transfection. (A) Immunostaining of FLAG‐Praja and tau in SH‐SY5Y cells transfected with FLAG‐tagged Praja1/Praja2 and tau alone or co‐transfected (*n* = 3). DAPI was used for nuclear staining. Scale bar: 10 μm. Fluorescence intensity profiles with Kullback–Leibler divergence along the indicated lines are plotted on the right. (B) Western blot analysis and quantification of FLAG‐tagged Praja1/Praja2 and tau co‐expressed in SH‐SY5Y cells showing reduced tau protein levels (*n* = 4). (C) Western blot analysis following 50 pmol Praja1 siRNA treatment on 35 mm dish, demonstrating a significant increase in tau protein levels (*n* = 3). (D) No decrease in endogenous tau was observed when Praja1 overexpressed (*n* = 3). (E) Treatment with 10 μM MG132 does not affect exogenous 2N4R tau levels (*n* = 3). (F) Western blotting for tau in SH‐SY5Y cells treated with or without 10 μm MG132 for 6 h and co‐expressed with Praja1. Quantification indicates inhibition of decrease in tau level by MG132 (*n* = 3). (G) Graphical representation of the RING finger domain of Praja1 (blue) and alanine mutated residues in the zinc finger motif (red). (H) Co‐expression experiments with RING domain‐deleted Praja1 (dPraja1) and tau showing no reduction in tau protein levels (*n* = 3). (I) Co‐expression of Praja1 mutated in the zinc finger motif of the RING finger domain (mPraja1) and its western blot analysis indicate Praja1's E3 ligase activity is required for tau protein reduction (*n* = 3). For statistical analyses more than 3 groups, mean values were subjected to multiple testing by analysis of variance with *post hoc* Dunnett test (n.s. *P* ≥ 0.10, *P < 0.10, **P < 0.05, ***P < 0.01). For 2 groups, mean values were subjected to Student's *t*‐test (n.s. *P* ≥ 0.10, *P < 0.10). Error bars indicate standard deviation. Error bars in the quantified immunofluorescence images were calculated using standard error.

As the Praja family regulates genes associated with neurodegenerative diseases and their products, we hypothesized that they could potentially interact with tau, which is also closely related to neurodegenerative diseases. To verify this, tau was expressed together with Praja family proteins in human neuroblastoma SH‐SY5Y cells, and the protein levels were biochemically analyzed. Tag‐free 2N4R tau and N‐terminal FLAG‐tagged Praja1/Praja2 were used. First, we verified that both the tau and Praja family proteins were expressed in the cytoplasm (Fig. 1A). Three 50px areas were randomly selected and Kullback–Leibler divergence was used to evaluate whether the fluorescence intensity correlates across these areas. The results showed that the fluorescent intensity in Praja1 remained almost the same, while Praja2 showed a different trend. Subsequently, co‐expression with Praja1 resulted in a statistically significant reduction in tau protein levels; however, no such effect was observed with Praja2 (Fig. 1B). Consistent with this result, targeted knockdown of Praja1 using siRNA resulted in an increase in the levels of endogenous tau (Fig. 1C), indicating that Praja1 levels are correlated with tau protein levels. However, overexpression of Praja1 did not result in a decrease in endogenous tau protein levels (Fig. 1D). This asymmetric response warrants further investigation, potentially focusing on differences in post‐translational modification and/or isoforms between endogenous and exogenous tau. In this study, the reduction in endogenous tau levels in SH‐SY5Y cells, which express all six isoforms, may not be observable, even if Praja1 can specifically recognize tau isoforms [17].

Next, we examined whether the reduction in tau levels depends on the E3 ligase activity of Praja1. First, the cells were treated with MG132 for 6 h to inhibit proteasomal degradation, and it was confirmed that MG132 does not alter tau levels (Fig. 1E). MG132 treatment restored tau protein levels, which were otherwise reduced by Praja1 (Fig. 1F). Furthermore, the RING domain of Praja1, which is essential for its E3 ligase activity, was deleted (dPraja1) or mutated (mPraja1) at the zinc finger residues (Fig. 1G) [18]. Expression of dPraja1 did not lead to a decrease in tau levels (Fig. 1H). Similarly, C613A/H615A mutations were sufficient to prevent the decrease in tau levels (Fig. 1I). These results suggest that Praja1 negatively regulates tau stability in SH‐SY5Y cells in an E3 ligase activity‐dependent manner.

### Interaction between Praja1 and tau

**Fig. 2:**
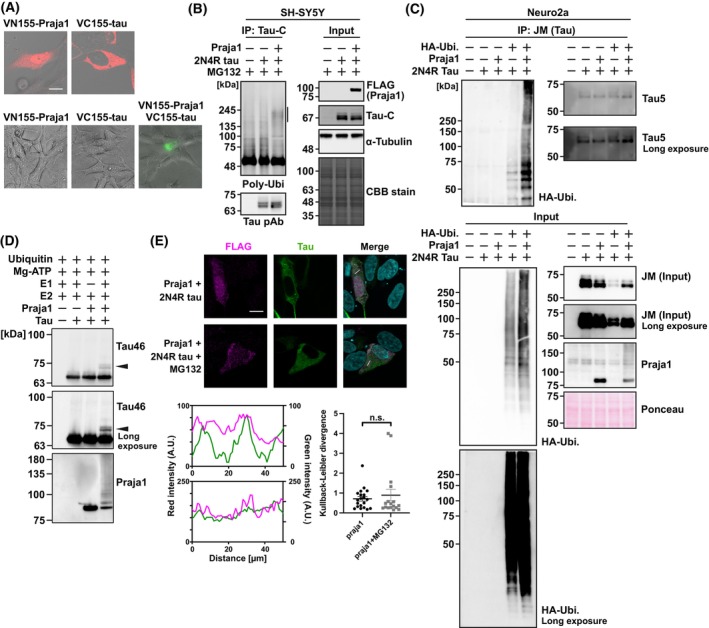
Praja1 may interact and ubiquitinate tau as a substrate. (A) Top panels: VN155‐Praja1 and VC155‐tau were solely transfected and confirmed its distribution within SH‐SY5Y cells. Bottom panels: VN155‐Praja1 and VC155‐tau co‐expressed in the SH‐SY5Y cells and Venus‐based bimolecular fluorescence complementation (BiFC) fluorescence was observed by fluorescence microscopy (*n* = 3). Scale bar: 10 μm. Fluorescence was only observed upon co‐transfection indicating two proteins are in proximity. (B) FLAG‐Praja1 and tau co‐expressed in the SH‐SY5Y cells treated with MG132 for 6 h. Immunoprecipitation was performed with tau‐C antibody, and multi‐ubiquitin was detected by Western blotting showing increased ubiquitination level in Praja1‐dependent manner. Vertical line shows potential ubiquitinated tau (*n* = 3). (C) Immunoprecipitated fraction using anti‐tau (JM) was analyzed by HA antibody in Neuro2a cells, resulting in Praja1 dependent increase in ubiquitination (*n* = 3). (D) *In vitro* ubiquitination assay was performed for 1 h, 37 °C using E1, E2, His‐Praja1, His‐tau, Ubiquitin, and Mg‐ATP (*n* = 3). (E) Immunostaining for FLAG‐Praja and tau in SH‐SY5Y cells transfected with both FLAG‐Praja1 and tau, treated with MG132 for 6 h. DAPI was applied for nuclear staining (*n* = 3). Scale bar: 10 μm. Fluorescence intensity profiles along the indicated lines are plotted at the bottom. For statistical analyses, mean values were subjected to Student's *t*‐test (n.s. *P* ≥ 0.10). Error bars indicate standard deviation. Error bars in the quantified immunofluorescence images were calculated using standard error.

Based on the above results, we hypothesized that tau is a substrate of Praja1. First, a Venus‐based bimolecular fluorescence complementation (BiFC) assay was used to visualize the interaction between the two proteins in live cells [19]. We generated fusion proteins by attaching the C‐terminal (VC155) fragment of Venus to the N terminus of tau, creating VC155‐tau. Additionally, we generated VN155‐Praja1 by fusing VN155 to the N terminus of Praja1. Immunofluorescence analysis confirmed that these BiFC constructs exhibited subcellular localization similar to FLAG‐Praja1 and 2N4R‐tau (Fig. 2A). Co‐expression of VN155‐Praja1 with VC155‐tau resulted in cytoplasmic fluorescence, indicating proximity between these proteins. This fluorescence was not observed when each plasmid was transfected individually. Conversely, co‐expression of VN155‐Praja1 with VC155 fused at the C terminus of tau failed to generate fluorescence (data not shown). Next, the direct interaction between Praja1 and tau was examined using a His‐tag pull‐down assay. First, N‐terminal His‐tagged Praja1/tau was purified from *E. coli* (Fig. S1A–C). Surprisingly, tau did not exhibit any direct interaction with His‐Praja1 under our experimental conditions, indicating that the interaction between Praja1 and tau may be weak and transient (Fig. S1D).

We next investigated whether Praja1‐dependent ubiquitination of tau occurs under *in vivo* and *in vitro* conditions. An *in vivo* ubiquitination assay was conducted, and Praja1‐dependent polyubiquitination of tau was observed in cells expressing Praja1 and tau (Fig. 2B). This was confirmed not only in SH‐SY5Y cells but also in Neuro2a cells that constantly express HA‐ubiquitin (Fig. 2C). Next, we purified His‐tau from *E. coli* (Fig. S1E,F). His‐Praja1 and His‐tau were incubated with E1 (UBE1), E2 (UBE2D3), ubiquitin, and ATP—components necessary for the ubiquitin transfer reaction—to perform an *in vitro* ubiquitination assay. Consequently, putative monoubiquitinated tau appeared 5–10 kDa above His‐tau, which was absent in the absence of Praja1 or E1 (Fig. 2D). Additionally, the expression of both Praja1 and tau was confirmed in the cytoplasm following MG132 treatment, as some E3 ligases tend to relocate to aggresome structures (Fig. 2E) [20]. Taken together, these findings suggest that tau is a potential substrate of Praja1.

### An evolutionary deletion specific to Praja1 is required for tau recognition

**Fig. 3:**
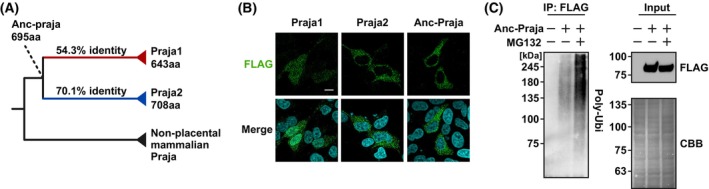
Resurrected ancestral Praja possess E3 ligase activity. (A) Ancestral Praja positions (Anc‐Praja) at common ancestor of Praja1 and Praja2. Anc‐Praja was reconstructed by graphical representation of ancestral sequence predictions (GRASP) software. (B) Immunostaining of FLAG‐Praja transfected into SH‐SY5Y cells shows cytoplasmic distribution of Anc‐Praja. DAPI was used for nuclear staining (*P* = 3). Scale bar: 10 μm. (C) FLAG‐Anc‐Praja and tau co‐transfected SH‐SY5Y cells were treated with MG132 for 6 h. Immunoprecipitation was performed using FLAG antibody, and multi‐ubiquitin was detected by Western blotting showing increased ubiquitin level in Anc‐Praja dependent manner (*n* = 3).

Next, we sought to investigate the evolutionary mechanism underlying the interaction between tau and Praja1. To address this, we reconstructed the common ancestral sequences of Praja1 and Praja2 (Anc‐Praja), which are common ancestors of the placenta (Fig. 3A), using graphical representations of ancestral sequence predictions (GRASP) [21].

Anc‐Praja showed 54.3% and 70.1% similarity to Praja1 and Praja2, respectively, with lengths of proteins in the following order: Praja1 (643 aa) < Anc‐Praja (695 aa) < Praja2 (708 aa). Sequence conservation between Anc‐Praja, Praja1, and Praja2 was relatively high in the N‐ and C‐terminal regions and low in the regions between them (Fig. S2). We expressed each Praja protein in SH‐SY5Y cells and observed that Praja1 was expressed in both the cytoplasm and nucleus, whereas Praja2 and Anc‐Praja were expressed only in the cytoplasm (Fig. 3B). This may be explained by the acquisition of a nuclear localization signal specific to Praja1 [2]. Notably, E3 ligases underwent auto‐ubiquitination during their activity [22, 23]. MG132 treatment increased the ubiquitinated protein levels in the Anc‐Praja immunoprecipitates. Given that Anc‐Praja possesses a RING domain that is highly similar to that of Praja1/Praja2 (40 out of 41 residues are identical between Anc‐Praja and Praja2), these ubiquitination signals may reflect either auto‐ubiquitination of Anc‐Praja itself and/or ubiquitination of its direct/indirect target proteins (Fig. 3C). These results suggest that the reconstructed Anc‐Praja has the potential to function as an E3 ligase, with sequence and localization more similar to Praja2 than to Praja1.

**Fig. 4:**
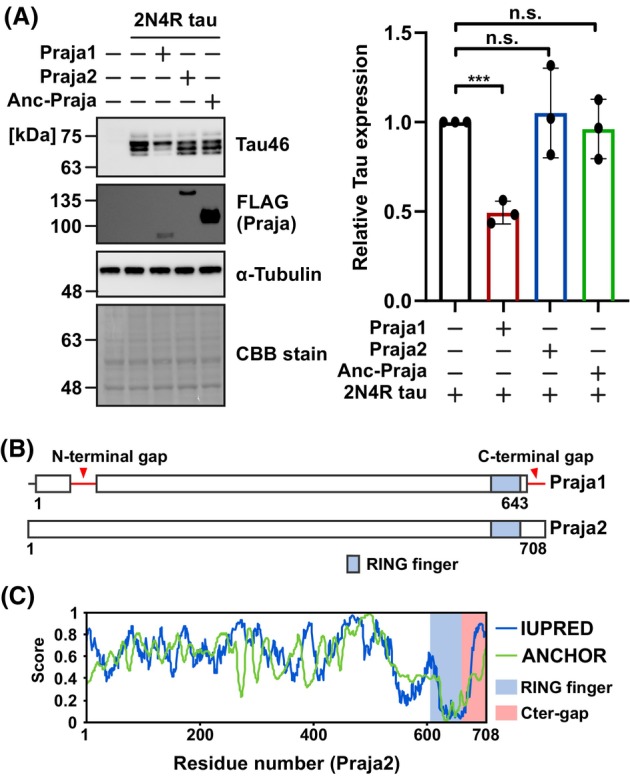
Praja1 may have started recognizing tau as a novel substrate during its evolution. (A) FLAG‐Praja and tau were co‐expressed in SH‐SY5Y cells and western blotted. Quantification indicates reduction of tau protein level when co‐expressed with Praja1 but not with Praja2 or Anc‐Praja (*n* = 3). The FLAG panel is derived from the same blot as shown in Fig. 1A using identical samples, with the addition of Anc‐Praja. Other panels represent independent blots from the same sample set, recaptured at different time points. (B) Schematic alignment of Praja1 and Praja2. Red arrows indicate regions lost during Praja1 evolution. Blue indicates the RING finger domain. (C) Analysis of disordered regions of Praja2 by IUPRED (blue line) and ANCHOR (green line). The blue region represents the RING finger and the red region represents the C‐terminal gap. For statistical analyses, mean values were subjected to multiple testing by analysis of variance with *post hoc* Dunnett test (n.s. *P* ≥ 0.10, ***P < 0.01). Error bars indicate standard deviation.

Anc‐Praja and tau were co‐expressed in SH‐SY5Y cells. However, no Anc‐Praja‐dependent reductions in tau protein levels were observed (Fig. 4A). This suggests that Praja1 uniquely acquired the tau recognition ability during its evolution. As Praja1 underwent deletions in the N‐ and C‐terminal regions after duplication from Praja2 (Fig. 4B), we explored whether the regions are functional. The C‐terminal region (682–707 aa) is a widely conserved 26 amino acid region within the Praja2 sequences and is predicted to be an intrinsically disordered region, as shown by two disorder prediction programs (Fig. 4C) [24]. Interestingly, two missense SNPs have been reported in humans (rs139484791: A705T and rs246105: A688D/V) [25, 26, 27]. Notably, rs246105 had a higher allele frequency in a group of non‐small‐cell lung cancer patients with recurrence within three years after surgery, suggesting that the corresponding region plays a significant physiological role. These results suggest that the C‐terminal region of Praja2 may explain the functional divergence between Praja1 and Praja2 although experimental validation is crucial.

### Praja1 interacts with FTDP‐17‐specific variant P301L tau

**Fig. 5:**
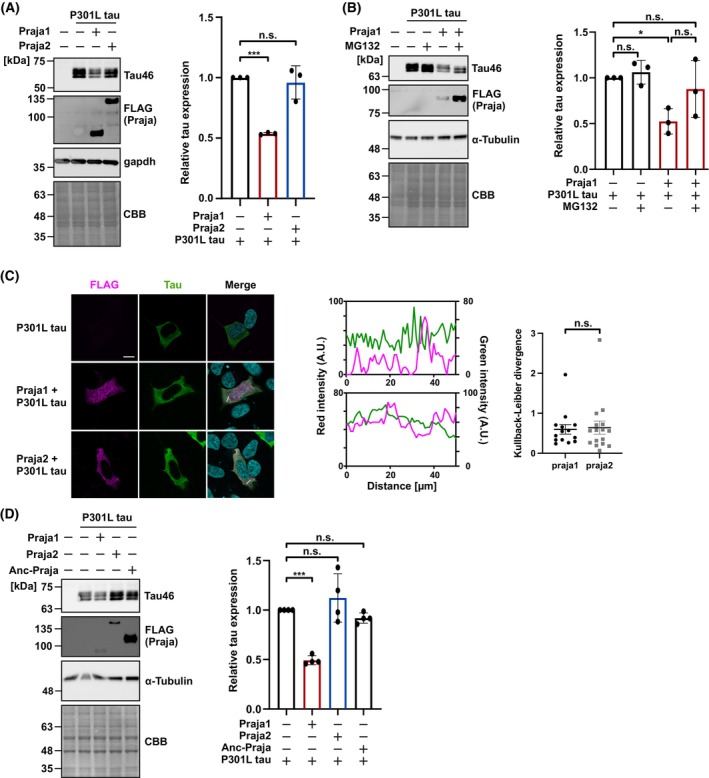
Praja1 recognizes P301L tau in SH‐SY5Y cells. (A) FLAG‐tagged Praja1/Praja2 and P301L tau were co‐expressed in SH‐SY5Y cells and western blotted. Quantification implied reduction of P301L tau by co‐transfection of Praja1 (*n* = 3). (B) FLAG‐tagged Praja1 and P301L tau were co‐expressed in SH‐SY5Y cells treated with or without MG132 for 6 h and western blotted showing tau reduction is inhibited by MG132 (*n* = 3). (C) Immunostaining of FLAG‐Praja and tau in SH‐SY5Y cells transfected with FLAG‐tagged Praja1/Praja2 and P301L tau. DAPI was used for nuclear staining. Scale bar: 10 μm (*n* = 3). Fluorescence intensity profiles along the indicated lines are plotted on the right. (D) FLAG‐Anc‐Praja and tau were co‐expressed in SH‐SY5Y cells and western blotted indicating no decrease of tau protein level upon Anc‐Praja cotransfection (*n* = 4). For statistical analyses more than three groups, mean values were subjected to multiple testing by analysis of variance with *post hoc* Dunnett test (n.s. *P* ≥ 0.10, *P < 0.10, ***P < 0.01). For 2 groups, mean values were subjected to Student's *t*‐test (n.s. *P* ≥ 0.10). Error bars indicate standard deviation. Error bars in the quantified immunofluorescence images were calculated using standard error.

To understand the pathological significance of tau degradation by Praja1, we focused on the P301L mutant among the missense variants often found in patients with FTDP [11, 28, 29, 30]. P301L tau is known to be excessively phosphorylated, aggregated, and to interact abnormally with its binding partners under disease conditions [31, 32]. Therefore, each Praja family member was tested for its effect on the P301L mutation. P301L tau was transiently expressed in SH‐SY5Y cells alone or together with Praja1 or Praja2. Praja1 reduced the expression of P301L tau, whereas Praja2 had no effect (Fig. 5A). To determine whether P301L tau was degraded by the E3 activity of Praja1, the cells were treated with MG132 for 6 h. Treatment with MG132 restored tau expression, which was suppressed by Praja1 (Fig. 5B). Furthermore, Praja1 co‐expressed with P301L tau was localized to the cytoplasm of SH‐SY5Y cells (Fig. 5C). Additionally, Anc‐Praja did not degrade P301L tau or WT tau. These results suggest that P301L tau and WT tau are substrate candidates of Praja1 (Fig. 5D). Together, these data show Praja1 potentially degrades P301L tau similar to WT tau.

## Discussion

In this study, we identified tau as a potential substrate of Praja1 and discerned the regions evolutionarily involved in its interaction and relationship with the P301L tau mutant. Notably, tau was degraded only by the Praja1 paralog through its E3 ligase activity. Although direct binding was not observed between Praja1 and tau using pull‐down assay, BiFC‐based fluorescent and *in vivo*/*in vitro* tau polyubiquitination induced by Praja1 interaction was observed, indicating that tau is a candidate substrate of Praja1. Further, Anc‐Praja, the common ancestral sequence of Praja1 and Praja2, was reconstructed, and we showed that it could not degrade tau, indicating that Praja1 uniquely acquired its tau recognition ability during its evolution after the duplication event. Notably, this conserved region in Praja2 harbors SNPs that may be associated with disease risk in humans, suggesting that it is a functional region. Importantly, the disease‐causing P301L tau, which often interacts abnormally with its binding partners, was degraded by Praja1.

In the present study, we were unable to confirm a direct interaction between Praja1 and tau, although the two proteins may be in close proximity, as suggested by the results of the *in vivo*/*in vitro* ubiquitination assays. This could be partially explained by the intrinsically disordered nature of Praja1, which is generally considered difficult to detect using co‐IP or pull‐down assays that require a certain level of stable complex formation [33]. In addition, despite the increase in tau levels observed in siPJA1 cells, we did not observe a reduction in endogenous tau upon Praja1 overexpression. This discrepancy may be attributed to homeostatic mechanisms that rapidly replenish endogenous tau levels upon degradation, although this apparent contradiction requires further investigation. Also, endogenous tau protein expression levels are considerably lower than overexpression systems, creating technical limitations that prevented reliable detection of tau ubiquitination changes upon Praja1 silencing and demonstration of the reverse regulatory effect.

*In vitro* ubiquitination assay showed tau monoubiquitination by Praja1. It has been reported that a subset of proteins in humans can be targeted for proteasome‐dependent degradation through monoubiquitination [34]. Therefore, it is possible that Praja1‐mediated monoubiquitination could directly promote tau degradation. While monoubiquitin potentially mediates degradation of tau, we also observed polyubiquitination of tau in cells (Fig. 2B, C). This suggests that the monoubiquitination observed *in vitro* may serve as an initial step leading to polyubiquitin chain formation through E4 ubiquitin ligases that facilitate ubiquitin chain elongation using monoubiquitinated proteins as substrates [35, 36].

Here, Praja1 was shown to recognize tau as a possible substrate protein. Tau hyperphosphorylation and aggregation are associated with several neurodegenerative diseases. This finding supports previous reports that Praja1 functions as a common suppressor of the aggregation of neurodegenerative disease‐related proteins [7, 8, 9]. In contrast, Praja2, which has been previously reported to be downregulated in patients with AD, did not functionally reduce tau at the protein level in this study [10]. The sequence identity between Praja1 and Praja2 diversified after gene duplication, especially at positions 100–500 of Praja2 [2]. Notably, no specific region responsible for the Praja1‐tau interaction was identified in the present study.

The reason why Praja1 can bind to and inhibit the aggregation of multiple neurodegenerative disease‐associated proteins, including tau, remains unresolved. This may be explained by the intrinsically disordered nature of Praja1, lacking a specific tertiary structure. E3 ligases containing disordered regions adopt suitable conformations in a partner‐dependent manner [37, 38]. In addition to the characteristics of Praja1, the biochemical properties of interacting proteins may also facilitate the recognition by Praja1. While other E3 ligases are sequestered from protein aggregates (e.g., polyQ and TDP‐43 aggregates), Praja1 seems to successfully interact with aggregates for its clearance [7, 8, 9]. Further studies are needed to reveal the molecular mechanism by which Praja1 senses aggregated proteins and their pathological significance.

Tau is a disordered protein that flexibly adopts a specific conformation when interacting with microtubules [39, 40]. Phosphorylation of tau weakens its binding to microtubules, and free tau may lead to pathological aggregation [31, 41]. Most autosomal dominant FTLD‐tau mutations, including the P301L tau mutation used in this study, occur in the microtubule‐binding domain, resulting in accelerated phosphorylation of tau owing to altered interactions with microtubules [11, 27, 28, 29, 42]. Additionally, the mutations may alter the interactions with tau‐binding proteins [39]. Despite this mutation, Praja1 promoted the degradation of tau. Further investigation of the Praja1‐tau interaction is necessitated, as tau has diverse proteoforms, including splice variants and post‐translational modifications, perhaps using the E3 ligase CHIP, which specifically recognizes phosphorylated tau [43]. It is also not possible to discuss the differences among the six isoforms of human tau. Especially, as 2N4R tau was used in this study, the effect of N region deletion on interaction with Praja1 needs to be elucidated. In conclusion, our study on the regulation of tau proteostasis by Praja1 may provide insights into the pathogenesis of tauopathy.

## Materials and methods

### Plasmid construction

pCMV6‐Entry plasmid vectors encoding FLAG‐tagged human Praja1 and Praja2 were previously constructed [2]. pCMV‐2N4R tau was purchased from Cosmo Bio, Tokyo, Japan. The HA‐tagged Ubiquitin construct used in this study was previously reported [44]. Site‐directed mutations were generated using a KOD‐Plus‐Mutagenesis Kit (#SMK‐101; TOYOBO, Osaka, Japan). Each reaction was performed according to the manufacturer's instructions. Plasmids were sequenced to confirm their DNA sequences. The following primers were used for each construct:

C613A, H615A Praja1;

5′‐GCCCACGCCTATTTCCACAAGCCGTGTG‐3′.

5′‐CGGCAGCTCAGTTGCCACCTC‐3′.

P301L tau;

5′‐CGGGAGGCGGCAGTGTGCAAATAG‐3′.

5′‐GGACGTGTTTGATATTATCC‐3′.

### Cell culture and gene transfection

SH‐SY5Y cells (RRID: CVCL_0019; #94030304, European Collection of Authenticated Cell Cultures (ECACC), Porton Down, UK) were cultured in low‐glucose Dulbecco's modified Eagle's medium (DMEM #041–29 775; Wako Chemicals, Richmond, VA, USA) supplemented with 10% fetal bovine serum and 1% penicillin–streptomycin at 37 °C under 5% CO_2_. Neuro2A cells (RRID: CVCL_0470; #IFO50081, Japanese Collection of Research Bioresources Cell Bank (JCRB), Osaka, Japan) were cultured at 37 °C under 5% CO_2_ in high‐glucose DMEM supplemented with 10% fetal bovine serum (BioWest, France) and 1% penicillin–streptomycin (Nacalai tesque, #08458–16; Kyoto, Japan). All experiments were performed with mycoplasma‐free cells. For proteasome inhibition, SH‐SY5Y cells were incubated with 10 μm MG132 (#3175‐v; Peptide Institute Inc., Osaka, Japan) dissolved in DMSO. Plasmid vectors were introduced using Polyethyleneimine Max (#24765–100; Polysciences, Warrington, PA, USA) or Lipofectamine 3000 (#L3000008; Thermo Fisher Scientific, Waltham, MA, USA), and experiments were conducted 16–24 h post‐transfection. siRNA Praja1 was purchased from Thermo Fisher Scientific (#131786). Lipofectamine 3000 was used to transfect the gene into Neuro2A cells. Experiments were performed according to the manufacturer's protocol. The medium was changed 6–12 h after gene transfer and was used for experiments 48 h after gene transfer.

### Antibodies

Anti‐DYKDDDDK tag (#66008‐4‐IG; Proteintech, Rosemont, IL, USA), Anti‐HA (Mouse IgG1‐κ) HA124 (Nacalai tesque, #06340–96), Anti‐Rabbit IgG (H + L) (#711–035‐152; Wako) and Anti‐Mouse IgG Fcγ Fragment Specific (#115–035‐008, Wako), Anti‐Praja1 (#17687‐1‐AP; Proteintech), HRP‐conjugated anti‐mouse IgG (H + L) (#SA00001‐1; Proteintech), anti‐tau‐C (#TIP‐TAU‐P04; Cosmo Bio, Tokyo, Japan), anti‐tau‐360‐380 (#TIP‐TAU‐P02; Cosmo Bio), anti‐tau (#10274‐1‐AP; Proteintech), anti‐tau (RTM38, #017–26 893; Wako), anti‐tau (Tau5, # AHB0042; Thermo Fisher Scientific), anti‐tau (Tau pAb, #10274‐1‐AP; Proteintech) and anti‐tau (JM) [45] were used. Tau (Tau46) mouse monoclonal antibodies (4019) were purchased from Cell Signaling Technology, Danvers, MA, USA. Anti‐DDDDK‐tagged pAbs (anti‐FLAG, PM020), anti‐multi‐ubiquitin mAbs (FK2, D058‐3), and anti‐His‐tagged mAbs (OGHis) were purchased from MBL, Japan. Anti‐mouse IgG (H + L) Alexa Fluor™ 488 conjugated (R37114), goat anti‐rabbit IgG (H + L) cross‐adsorbed secondary antibody, Alexa Fluor^TM^ 594 (A11037) were purchased from Thermo Fisher Scientific. Phospho‐tau (Ser202 and Thr205) antibodies (AT8, MN1020) were purchased from Invitrogen, Waltham, MA, USA. Anti‐FLAG‐tag mAb (F1804‐200UG) was purchased from Sigma‐Aldrich, St. Louis, MO, USA.

### Western blotting and immunoprecipitation

SH‐SY5Y cells were lysed in cell lysis buffer (50 mm Tris–HCl (pH 7.8), 150 mm NaCl, 1% NP‐40, and 0.5% sodium deoxycholate) supplemented with a complete protease inhibitor cocktail (#11836153001; Roche, Basel, Switzerland). The cell lysate was centrifuged at 15000 ***g*** for 5 min at 4 °C, and the supernatant was treated with sample buffer solution (#198–13 282; Wako Chemicals) and 3% 2‐mercaptoethanol for 2 min at 95 °C. Protein concentrations were measured using a Pierce BCA Protein Assay Kit (#23225; Thermo Fisher Scientific) following the manufacturer's protocol. For SDS/PAGE, Extra PAGE One Precast Gel 7.5% (#13070–74; Nacalai, Kyoto, Japan) was used, and the proteins were subsequently transferred to a PVDF membrane (#IPVH00010; Merck Millipore, Burlington, MA, USA). The membranes were washed with TBS‐T (20 mm Tris (pH 7.5), 500 mm NaCl, and 1% Tween20) for 5 min and blocked with TBS‐T + 5% skim milk for 30 min at room temperature. The PVDF membrane was then incubated overnight with a primary antibody solution diluted in 2.5% skim milk and incubated for at least 1 h with an HRP‐conjugated secondary antibody solution diluted in 2.5% skim milk. Then, the PVDF membrane was treated with the chemiluminescence reagent Immunostar LD (#292–69 903; Wako Chemicals) for 10 s to 1 min, and images were acquired using a ChemiDoc Touch MP (Bio‐Rad, Hercules, CA, USA). The same membrane was subjected to CBB staining (#SP‐4011; APRO Science, Tokushima, Japan) for the quantitative evaluation of total proteins. The results were quantified using ImageJ and statistically analyzed using graphpad prism (GraphPad Prism 8 for Windows, graphpad Software, Boston, MA, USA, www.graphpad.com).

For immunoprecipitation, proteins were extracted as described above, and protein G MagSepharose (#28951379; Cytiva, Marlborough, MA, USA) conjugated with tau‐C antibody was used. Protein G MagSepharose and tau‐C antibodies were added and incubated at room temperature for 30 min. Protein extracts were then mixed at room temperature for 30 min, followed by the addition of sample buffer, and elution of bound proteins was performed at 95 °C for 5 min.

Neuro2A cells were washed with phosphate‐buffered saline (PBS‐) at pH 7.4 after a total of 2.5 μg overexpression of tau, HA‐Ubiq., Flag‐Praja1, and empty vector according to the previous section, then washed with leupepsin sulfate, Pepstatin A, 400 mm Na3VO4, 1 M NaF, 1 mm okadaic acid, aprotinin, modified RIPA Buffer (50 mm Tris–HCl (pH 7.4), 1% NP‐40, 0.25% NaDOC, 150 mm NaCl, 1 mm EGTA) containing 1 M glycerol phosphate. Centrifugation was performed at 16,000 ***g***, 4° for 10 min. 1 μL of tau antibody (JM) was added to the supernatant and rotated o/n at 4 °C for antibody reaction. Then, 5 μL per tube of FG Protein G (TAS8548N1173) was added and incubated at 4 °C for 2 h, washed four times with modified RIPA Buffer, and eluted with 1×sample buffer to prepare samples for SDS/PAGE.

Equal amounts of protein lysate were separated on SuperSepTM Ace 5~20% (#194–15 021; Wako) gels. The samples were transferred to a nitrocellulose blotting membrane Amersham Protran 0.2 μm NC (#10600001; Cytiva). The membranes were Ponceau stained and blocked by treatment with a 5% skim milk solution in PBS‐T solvent for 1 h at room temperature. The membranes were then incubated overnight with primary antibody solution diluted in 3% BSA solution. Subsequently, the membranes were incubated with a secondary antibody solution conjugated with HRP diluted in 3% BSA solution for at least 2 h. The samples were treated with chemiluminescent reagent Chemi‐Lumi One L (#07880–70; Nacalai tesque) or ECL Prime WB Detection R (#RPN2232; Cytiva) for 1.5 min and images were acquired on an imaging system AI600.

### Immunofluorescence and microscopy

Immunofluorescence analysis was performed as previously described [2]. The SH‐SY5Y cells were washed with PBS and fixed in 4% paraformaldehyde for 20 min at room temperature. The fixed cells were permeabilized with 0.3% Triton X‐100 in PBS for 10 min at room temperature. The permeabilized cells were blocked with 5% BSA/PBS for 30 min at room temperature. Cells were then incubated overnight at 4 °C with the primary antibody dissolved in 2.5% BSA/PBS. Cells were incubated with the same solvent and secondary antibody for 2 h at 4 °C. Cell nuclei were stained with Vectashield mounting medium for fluorescence using DAPI (#H‐1200; Vector Laboratories Inc., Newark, CA, USA). Images were captured using a confocal laser scanning microscope (#FV‐3000; Olympus, Tokyo, Japan).

### BiFC assay

To investigate the protein–protein interactions between tau and Praja1, we employed the BiFC assay [19]. The Venus fluorescent protein (Ex515/Em528) split into two fragments, VN‐155 (I152L, 1–154 aa) and VC‐155 (155–239 aa), were fused to tau and Praja1 together with the linker sequence RSIAT. DNA sequences of Venus fragments with the linker sequence were synthesized in Eurofins Scientific, Louisville, KY, USA. VN‐155 (I152L) was fused to the N terminus of Praja1, and VC‐155 was fused to either the N terminus or C terminus of tau. We used N‐terminally tagged tau as C‐terminally tagged tau did not show Venus‐derived fluorescent (data not shown). For the subcloning of tau and Venus fragments into the pCMV6‐Entry vector, the HiFi Assembly Kit (NEB, #E2621) was utilized.

### *In vitro* ubiquitination assay

*In vitro* ubiquitination was performed to confirm whether Praja1 directly recognizes tau. For this purpose, 100 nm E1 (UBE1; #E‐305‐025, R&D systems), 1 μm E2 (UBE2D3; #E2‐627‐100, R&D systems), 2 μm His‐Praja1, 3 μm His‐tau, 100 μm ubiquitin (#U‐100H‐10 M, R&D systems), and 10 mm Mg‐ATP (#B‐20, R&D systems) were added to a total volume of 10 μL in phosphate buffer with 1 mm DTT. The reaction was conducted at 37 °C for 1 h. The E2, UBE2D3, was selected as it is known to co‐operate with Praja1 as an E3 ligase complex [46]. To stop the reaction, sample buffer containing 5% 2ME was added and heated at 95 °C for 2 min.

### Construction of ancestral Praja1

A common ancestor sequence of Praja1 and Praja2 was reconstructed using 202 previously collected mammalian Praja family sequences [2]. GRASP was used to reconstruct the ancestral sequence using the joint reconstruction option [21]. Using this option, the most probable state in the combination of all ancestral states was calculated by optimizing the likelihood over the entire phylogenetic tree. The amino acid sequence obtained by GRASP was codon‐optimized for *Homo sapiens*, and plasmid DNA was obtained by gene synthesis from Eurofins Scientific, Louisville, KY, USA.

### Protein purification and pull‐down assay

The human Praja1 sequence was ordered from Eurofins Scientific with codon optimization in *E. coli*. This sequence was inserted into the pET‐HisTEV vector and transformed into Rosetta2 (DE3) cells (#71397; Merck Millipore) to express N‐terminally His‐tagged Praja1. Colonies picked from the above were precultured in 100 mL of LB medium overnight and then grown in 2000 mL of medium for 1.5 h. When OD_600_ reached 0.6–0.8, His‐Praja1 expression was induced with 0.5 mm Isopropyl‐β‐D‐thiogalactopyranoside. The centrifuged pellet was resuspended in 1.5 mL of sonication buffer (50 mm Tris–HCl (pH 8.0), 150 mm NaCl, and a protease inhibitor) and subjected to ultrasonication. After centrifugation at 15000 ***g*** for 10 min at 4 °C, the supernatant was purified using a 0.45 μm PVDF filter and used as the input sample to be used for purification. Purification of this sample was performed using a HisTrap HP (5 mL, #17524802; Cytiva) on an Akta avant25 column (Cytiva). The column was equilibrated with 50 mm Tris–HCl (pH 8.0), 150 mm NaCl, and 50 mm imidazole, injected, and washed with the same buffer. For the elution of His‐Praja1, 50 mm Tris–HCl (pH 8.0), 150 mm NaCl, and 500 mm imidazole were used. The fractions containing His‐Praja1 were ultrafiltered, concentrated with 30 kDa NMWL Amicon Ultra (#UFC503024; Merck Millipore), and used for downstream analysis. We confirmed the presence of His‐Praja1 in the eluted fraction using SDS/PAGE and western blotting with an anti‐His tag. Additionally, 1 h of TEV protease treatment was used to verify whether the His‐tag was removed from Praja1 and to confirm that the purification was successful.

A pull‐down assay was performed using a Capturem His‐Tagged Purification Miniprep Kit (#635710; Takara, Shiga, Japan). 50 μg of BSA was added to the column for blocking, followed by 5 μg of purified His‐Praja1. The column was then centrifuged at 11,000 × ***g*** for 1 min. An extract of tau‐overexpressing SH‐SY5Y cells was applied to the column and centrifuged under the same conditions. The extract was then eluted with wash and elution buffers containing 500 mm imidazole. The eluted fractions were treated with sample buffer and 2‐mercaptoethanol for 2 min at 95 °C and used as samples for western blotting.

## Author contributions

SA, KK, KI, HK, MO, and WO performed the experiments with support from YS and AT. SA wrote the manuscript with support from WO and TA, who supervised and designed the project. Funding was acquired by WO and TA Resources were provided by WO, YS, SA, and TA All authors discussed the results and contributed to the final manuscript.

## Conflict of interest

The authors declare no conflict of interest.

## Supporting information


**Fig. S1.** Purification of His‐tagged Praja1 and pull‐down assay with tau.
**Fig. S2.** Multiple sequence alignment of human Praja1, Praja2 and ancestral Praja.

## Data Availability

The data supporting the findings of this study are available within the Supporting Information of this article.
