# Supplementary material for: E3 ligase Praja1 mediates ubiquitination and degradation of microtubule‐associated protein tau
Source: FEBS J. 2025 Nov 3;293(8):2212–24. doi: 10.1111/febs.70303 (PMC13080228; doi:10.1111/febs.70303)
Supplement: Supplementary file 1 — Fig. S1. Purification of His‐tagged Praja1 and pull‐down assay with tau. Fig. S2. Multiple sequence alignment of human Praja1, Praja2 and ancestral Praja. [file FEBS-293-2212-s001.pdf]

Supporting information for

E3 ligase Praja1 mediates ubiquitination and degradation of microtubule-associated protein tau

Shiho Aoki, Wataru Onodera, Akihiko Takashima, Kotaro Kawasaki, Kazuki Imadegawa, Hikaru Kurahashi, Mizuho Oishi, Toru Asahi, Yoshiyuki Soeda

Correspondence to: w.onodera@aoni.waseda.jp

Supporting information 1 to 2

Fig. S1. Purification of His-tagged Praja1 and pull-down assay with tau. A. Chromatography by HisTrap HP to purify His-tagged Praja1 using AKTA Avant. Grey highlighted region was used for further experiment after ultrafiltered and concentrated with 30 kDa NMWL Amicon Ultra. B. CBB staining of purified His-Praja1 with TEV protease. Recognition site for TEV protease exist in between His-tag and Praja1. C. Western blot for purified His-tagged Praja1 with TEV protease treatment shows successful cleavage of His-tag from Praja1. D. Western blot for tau from N-terminal His-tagged Praja1 purified from E. coli using pull-down assay indicating no interaction between His-Praja1 and tau protein. E. Chromatography by HisTrap HP to purify His-tagged 2N4R tau using AKTA Avant. Grey highlighted region implies fraction confirmed with His-tau protein. F. CBB staining shows successful purification of His-tau.

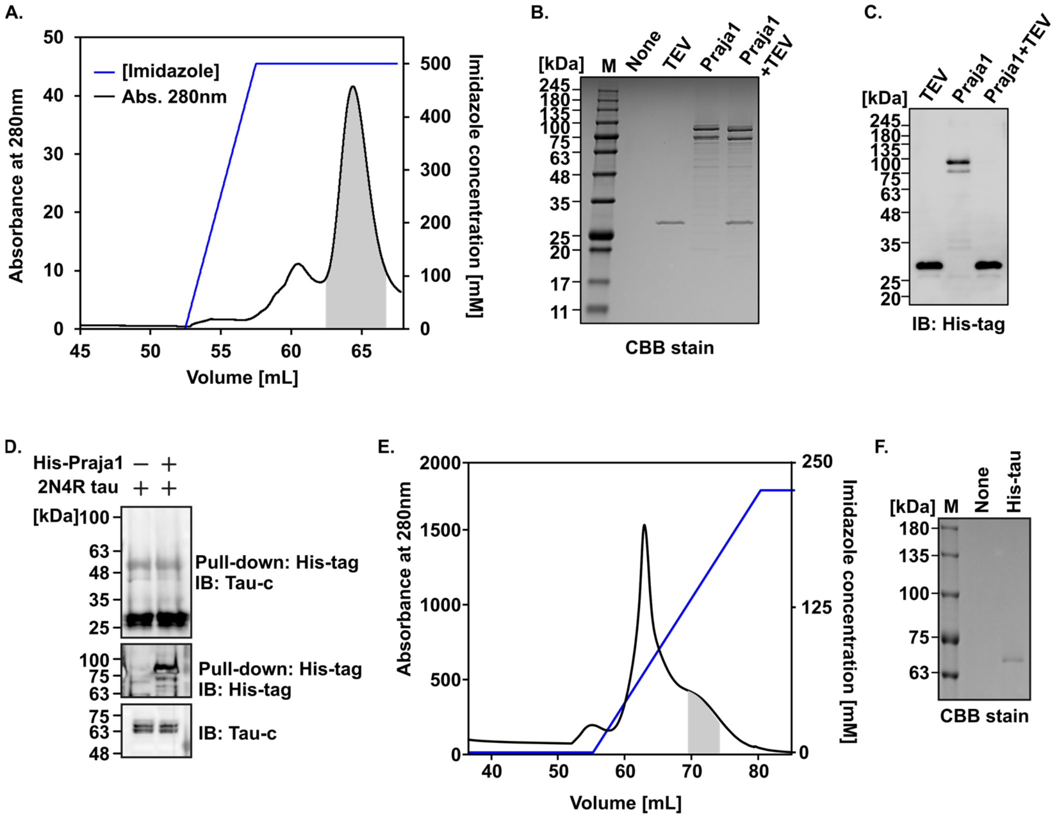

Fig. S2. Multiple sequence alignment of human Praja1 (GenBank accession number: NP_001369704.1), Praja2 (GenBank accession number: NP_055634.3) and ancestral Praja. Ancestral Praja was reconstructed at the common ancestor of placentals which corresponds to the ancestor sequence of Praja1 and Praja2. Sequence alignment was performed using ClustalW webserver (https://www.genome.jp/tools-bin/clustalw) under default parameters.

```
Anc_Praja                   -----------MGQESSKPTWPKPAGGYQTITGRRYGRRHAYVSFKPSMTRQERSSSQQS
Homo-sapiens_Praja2         MSQYTEKEPAAMDQESGKAVWPKPAGGYQTITGRRYGRRHAYVSFKPCMTRHERSLGRAG
Homo-sapiens_Praja1         -----------MGQESSKPVWPNPTGGYQSNTGRRYGRRHAYVSFRPPTSQRERIASQ--
                                       *.***.*..**:*:****: **************:*  :::**  .:

Anc_Praja                   GEYEGLELDDVQKEKASCCSPLDQVHPSLSGEPLLEKAGAEIPICRAALTQTTERSPSPF
Homo-sapiens_Praja2         DDYEVLELDDVPKENSSGSSPLDQVDSSLPSEPIFEKSETEIPTCGSALNQTTESSQS-F
Homo-sapiens_Praja1         -----------------------------------RKTNSEVPMHRSAPSQTTKRSRSPF
                                                               .*: :*:*   :* .***: * * *

Anc_Praja                   SAIHHSLEGSETSGSSLNLDNSSDSRYTPREYNASSVKNGIAFVHIDSYEPDGSDGEDNS
Homo-sapiens_Praja2         VAVHHSEEGRDTLGSSTNLHNHSEGEYIPGACSASSVQNGIALVHTDSYDPDGKHGEDND
Homo-sapiens_Praja1         STTRRSWDDSESSGTNLNIDNEDYSRYPPREYRASGSRRGMAYGHIDSYGADDSEEEGAG
                             : ::* :. :: *:. *:.* . ..* *    **. :.*:*  * *** .*... *. .

Anc_Praja                   QIELSAVKVETGKFQETLDDMLYDLEKG-VDSLSGLPPQFSSFNCEIREEFEELDPAPLV
Homo-sapiens_Praja2         HLQLSAEVVEGSRYQESLGNTVFELENREAEAYTGLSPPVPSFNCEVRDEFEELDSVPLV
Homo-sapiens_Praja1         PVERPPVRGKTGKFKDDK----LYDPEKGARSLAGPPPHFSSFSRDVREERDKLDPVPAA
                             :: ..   : .::::          :  . : :* .* ..**. ::*:* ::**..* .

Anc_Praja                   KYSSSDTEFVHQNNRTLQRSSAEDEVVAKKYQNNSSRERQRKNLTEDPARAPVDICNEQN
Homo-sapiens_Praja2         KSSAGDTEFVHQNSQEIQRS-SQDEMVSTKQQNNTSQERQTEHSPEDAACGPGHICSEQN
Homo-sapiens_Praja1         RCSASRADFLPQSSVASQSS---SEGKLATKGDSSERERREQNLPARPSRAPVSICG---
                            : *:. ::*: *..   * *   .*    .  :.:.:**: :: .  .: .*  **.

Anc_Praja                   TSDGEKNQGSSSELVVRPKVRKLASSSQVDQKTLFNSDEDK--QHSIERWRETSEAEENH
Homo-sapiens_Praja2         TNDREKNHGSSPEQVVRPKVRKLISSSQVDQETGFNRHEAK--QRSVQRWREALEVEESG
Homo-sapiens_Praja1         --GGENTSKSAEEPVVRPKIRNLASPNCVKPKIFFDTDDDDDMPHSTSRWRDTANDNEGH
                              . *:.  *: * *****:*:* *.. *. :  *: .: .   :* .***:: : :*.

Anc_Praja                   SDGLLR---KYDGENSSMFFDPRYS--EAQRETKNNQIKPETVTTQGRQAIVDNTFWNSC
Homo-sapiens_Praja2         SDDLLIKCEEYDGEHDCMFLDPPYSRVITQRETENNQMTSESGATAGR-QEVDNTFWNGC
Homo-sapiens_Praja1         SDGLAR---RGRGESSSGYPEPKYP--EDKREARSDQVKPEKVPRRRR-TMADPDFWTHS
                            **.*     .  **..  : :* *.    :**:..:*:..*. .   *   .*  **. .

Anc_Praja                   EDYYRYYDKDEDSSECSDGEWSASLPHRFSGREKDQSSSDESWETLPGKEEHEPE-----
Homo-sapiens_Praja2         GDYYQLYDKDEDSSECSDGEWSASLPHRFSGTEKDQSSSDESWETLPGKDENEPE-----
Homo-sapiens_Praja1         DDYYKYCDEDSDS----DKEWIAALRRKYRSREQTLSSSGESWETLPGKEEREPPQAKVS
                             ***:  *:*.**    * ** *:* ::: . *:  ***.*********:*.**

Anc_Praja                   ----------------------LQSNSSGPEEENQELSLQEGEQTSLEEGEIPWLQYNEE
Homo-sapiens_Praja2         ----------------------LQSDSSGPEEENQELSLQEGEQTSLEEGEIPWLQYNEV
Homo-sapiens_Praja1         ASTGTSPGPGASASAGAGAGASAGSNGSNYLEEVREPSLQE-EQASLEEGEIPWLQYHE-
                                                    *:.*.  ** :* **** **:************:*

Anc_Praja                   NESSSDEENDPANEFVQPGVFMLDGNNNLEDDSSVSEDLDVDWSLFDGFADGLGVAEAIS
Homo-sapiens_Praja2         NESSSDEGNEPANEFAQP-AFMLDGNNNLEDDSSVSEDLDVDWSLFDGFADGLGVAEAIS
Homo-sapiens_Praja1         NDSSSEGDNDSGHELMQPGVFMLDGNNNLEDDSSVSEDLEVDWSLFDGFADGLGVAEAIS
                            *:***:  *:..:*: ** .*******************:********************

Anc_Praja                   YVDPQFLTYMALEERLAQAMETALAHLESLAVDVEVANPPASKESIDCLPETLVTEDHTA
Homo-sapiens_Praja2         YVDPQFLTYMALEERLAQAMETALAHLESLAVDVEVANPPASKESIDGLPETLVLEDHTA
Homo-sapiens_Praja1         YVDPQFLTYMALEERLAQAMETALAHLESLAVDVEVANPPASKESIDALPEILVTEDHGA
                            *********************************************** *** ** *** *

Anc_Praja                   VGQEQCCPICCSEYIKDEIATELPCHHFFHKPCVSIWLQKSGTCPVCRHVFPPALPEATA
Homo-sapiens_Praja2         IGQEQCCPICCSEYIKDDIATELPCHHFFHKPCVSIWLQKSGTCPVCRRHFPPAVIEASA
Homo-sapiens_Praja1         VGQEMCCPICCSEYVKGEVATELPCHHYFHKPCVSIWLQKSGTCPVCRCMFPPPL-----
                            :*** *********:*.::********:********************  ***.:

Anc_Praja                   ATSFLPDHDSPPSIHSATGTQ
Homo-sapiens_Praja2         APSSEPDPDAPPSNDSIAEAP
Homo-sapiens_Praja1         ---------------------
```
